# Increased interactions and engulfment of dendrites by microglia precede Purkinje cell degeneration in a mouse model of Niemann Pick Type-C

**DOI:** 10.1038/s41598-019-51246-1

**Published:** 2019-10-11

**Authors:** Larisa Kavetsky, Kayla K. Green, Bridget R. Boyle, Fawad A. K. Yousufzai, Zachary M. Padron, Sierra E. Melli, Victoria L. Kuhnel, Harriet M. Jackson, Rosa E. Blanco, Gareth R. Howell, Ileana Soto

**Affiliations:** 1 Department of Molecular & Cellular Biosciences, Rowan University, Glassboro, NJ USA; 2 The Jackson Laboratory, Bar Harbor, ME USA; 3 The Institute of Neurobiology, University of Puerto Rico, San Juan, PR USA

**Keywords:** Neurodegeneration, Microglia, Cerebellum

## Abstract

Niemann Pick Type-C disease (NPC) is an inherited lysosomal storage disease (LSD) caused by pathogenic variants in the *Npc1* or *Npc2* genes that lead to the accumulation of cholesterol and lipids in lysosomes. NPC1 deficiency causes neurodegeneration, dementia and early death. Cerebellar Purkinje cells (PCs) are particularly hypersensitive to NPC1 deficiency and degenerate earlier than other neurons in the brain. Activation of microglia is an important contributor to PCs degeneration in NPC. However, the mechanisms by which activated microglia promote PCs degeneration in NPC are not completely understood. Here, we are demonstrating that in the *Npc1*^*nmf164*^ mouse cerebellum, microglia in the molecular layer (ML) are activated and contacting dendrites at early stages of NPC, when no loss of PCs is detected. During the progression of PCs degeneration in *Npc1*^*nmf164*^ mice, accumulation of phagosomes and autofluorescent material in microglia at the ML coincided with the degeneration of dendrites and PCs. Feeding *Npc1*^*nmf164*^ mice a western diet (WD) increased microglia activation and corresponded with a more extensive degeneration of dendrites but not PC somata. Together our data suggest that microglia contribute to the degeneration of PCs by interacting, engulfing and phagocytosing their dendrites while the cell somata are still present.

## Introduction

The pathophysiology of NPC includes liver dysfunction, splenomegaly, neurodegeneration, dementia, and early death^1,2^. The age of onset of the neurological disease can occur at infantile (early or late), juvenile or adult stage of life^3^. However, the majority of NPC cases are diagnosed at late infantile ages^3^. Early neurological symptoms such as clumsiness, vertical gaze palsy, and gait disturbances occur as a result of early cerebellar PCs degeneration^4^. However, patients with the late-onset NPC also present early symptoms of cognitive decline, ataxia, dystonia and the development of neuropsychiatric disorders that precede dementia and death^5,6^. Human NPC has been recapitulated in several mouse models where NPC1 deficiency leads to the degeneration of PCs^7–11^. Among these mouse models is also the *Npc1*^*nmf164*^ mouse, which carries a point-mutation (D1005G) in the *Npc1* gene that affects protein folding and lead to the protein degradation^9^. The *Npc1*^*nmf164*^ mice present a late-onset and slower disease progression that is more representative of the juvenile form of human NPC disease^5,9^. Since, the progression of neurodegeneration is delayed in the *Npc1*^*nmf164*^ mouse, this mouse model is ideal to study neuropathological events that precede neurodegeneration and are caused by the deficiency of the NPC1 protein.

It is known that in NPC, PCs die mainly by necroptosis (a programmed necrosis)^12^. The activation of the necroptosis RIPK3/RIPK1 signaling pathways in neurons and glia, lead to the generation of damage associated molecular patterns (DAMPs) and cytokines that promote inflammation^13–15^. The potential release of DAMPs, such as ATP gradients, can induce mechanisms of microglia chemotaxis and migration, promoting the eventual recognition of “eat me” signals in the membrane of necrotic or damaged neurons that promote phagocytosis. However, the phagocytosis of viable neurons, or the induction of death by phagocytosis of neuronal structures during neuroinflammation has been proposed before^16–18^. It is possible that the death of PCs by necroptosis in NPC promotes and amplifies neuroinflammation, which could accelerate the degeneration of stressed, but otherwise live neurons, during the progression of the disease. In fact, microglial activation has been strongly implicated in the pathogenesis of NPC^19–22^. A neuroinflammatory phenotype of microglia has been described as early as two weeks of age in *Npc1*^*nih*^ mice^23^, while expression changes in genes associated to immune responses in the cerebellum have been reported in these mice at wean age^24^. Treatment of *Npc1*^nih^ mice with the anti-inflammatory drug ibuprofen, decreased CD68^+^ phagosomes in microglia and prolonged the survival of these mice^22^. Ibuprofen can exert inhibitory effects on phagocytosis as demonstrated in different types of phagocytic cells, including microglia^25–27^. Recent *in vitro* studies, have shown that NPC1 deficient microglia have increased phagocytic activity when compared to wild type microglia^20^. Genetic inhibition of microglia activation in *Npc1*^nih^ mice, delays disease progression and increases mouse survival time by 15%^20^, suggesting that activated microglia in NPC could contribute to the degeneration of PCs by phagocytosing stressed or damaged cells.

Here, we investigated the timing and extent of PCs degeneration in relation to microglia activation, and the correlation of these phenotypes to motor deficits in the *Npc1*^*nmf164*^ mouse. Interestingly, we found that activated phagocytic microglia and interaction with PC dendrites precede PC loss. Our results suggest that increased interactions between microglia and PC dendrites contribute to PCs degeneration in NPC.

## Results

### Behavioral deficits in the *Npc1*^*nmf164*^ mouse are exacerbated by age

**Figure 1:**
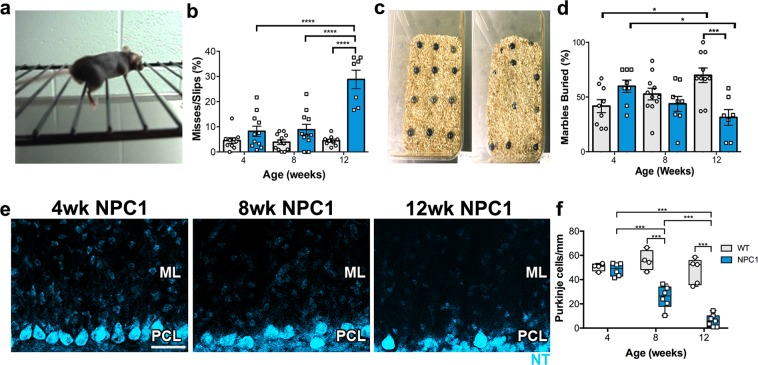
Motor deficits occur along severe loss of PCs in the *Npc1*^*nmf164*^ mouse. (**a**) Representative photograph illustrating a mouse in the ladder while one of its legs slipped between rungs. (**b**) Motor impairments in *Npc1*^*nmf164*^ mice when performing the ladder rung task at 4 (WT n = 10, NPC1 n = 11), 8 (WT n = 12, NPC1 n = 11), and 12 (WT n = 9, NPC1 n = 7) weeks of age. Quantifications of the percentage of misses and slips showed significant increases at 12 weeks of age in *Npc1*^*nmf164*^ mice when compared to WT mice. (**c**) Representative photograph illustrating the arrangement of marbles in the cage before (left) and after (right) the marble burying test. (**d**) Increased percentage of buried marbles at the early stage (4 wks) in *Npc1*^*nmf164*^ mice, while a significant decrease is observed at the severe stage of PCs degeneration (12 wks) when compared to WT mice (n = 7–10). (**e**) PCs stained with Neuro Trace^TM^ (NT) fluorescent nissl stain at 4, 8 and 12 weeks of age in *Npc1*^*nmf164*^ mice. (**f**) The linear density of PCs significantly decreased with age in regions from the first four lobules of the anterior cerebellum in *Npc1*^*nmf164*^ mice. No changes in the density of PCs were found between WT mice at 4, 8 and 12 weeks of age (n = 3–4 per age). Data are presented as mean ± SEM, 4 wk NPC1 (n = 6), 8 wk NPC1 (n = 6), 12 wk NPC1 (n = 7). *P < 0.05, ***P < 0.001, ****P < 0.0001. Scale bar: (**a**) 40 μm.

Since, abnormal and uncoordinated movements (ataxia) are hallmarks of cerebellar degeneration, to be able to determine asymptomatic, pre-symptomatic and symptomatic stages in the *Npc1*^*nmf164*^ mouse model, two behavioral tests that required motor coordination were performed at 4, 8 and 12 weeks (wks) of age. These ages were selected to cover the critical period of time of the disease onset and progression in the *Npc1*^*nmf164*^ mouse, which occur between post-weaning age and 14wks old, which is the average life-span of this mouse strain. First, wild-type (WT) and *Npc1*^*nmf164*^ mice were tested using the ladder rung walking task^28^ (Fig. 1a,b). The percentage of misses and slips, which included the failure to put the paw directly onto the rung, placing of the paws between the rungs or paws slipping off the rungs were calculated. Significant deficits were observed at 12 wks, but not at 4 and 8 wks of age, in *Npc1*^*nmf164*^ mice when compared to WT mice (Fig. 1b). Test performance was not different between sexes (Supp. Fig. S1a). To test how the *Npc1*^*nmf164*^ mutation affects species-typical spontaneous behaviors in mice, we used the marble burying test (Fig. 1c), which has been used previously to reflect alterations in motor activity and behaviors such as repetitive and perseverative behaviors^29,30^. In this test, only 16 (WT) and 28 (*Npc1*^*nmf164*^) percent of the females at 4 wks of age performed the test, while all the male and female mice at 8 and 12 wks engaged in the activity (Supp. Fig. S1b). Since the behavior of the 4 wks female mice was genotype independent but sex dependent, only mice that performed the test were included in the results. The number of buried marbles significantly increased with age in WT mice, whereas in *Npc1*^*nmf164*^ mice, significantly fewer marbles were buried at 12 wks of age when compared to 12 wks-WT and 4 wks-*Npc1*^*nmf164*^ mice (Fig. 1d). WT males were better than females at burying the marbles at 12 wks (Supp. Fig. S1c). Interestingly, 4 wks-*Npc1*^*nmf164*^ mice buried as many marbles as 12 wks-WT mice, suggesting a possible altered digging behavior in these mice. To further test this observation, we used the digging test^30^ to quantify the actions and duration of the digging behavior (Supp. Fig. S2a,b). Digging and wall rearing activity are normal mouse spontaneous behaviors in mice placed in empty cages. At 4 wks, the majority of the *Npc1*^*nmf164*^ mice showed higher digging activity and duration than WT mice (Supp. Fig. S1a,b). However, the duration of wall rearing was not different between 4 wks mice (Supp. Fig. S2c), suggesting a possible repetitive and perseverative behavior that is specific for the digging activity in young *Npc1*^*nmf164*^ mice. No differences of these behaviors were found between sexes (Supp. Fig. S2d,e). Overall, these results suggest that in *Npc1*^*nmf164*^ mice, loss of motor coordination is evident at 12 wks, making the 4 and 8 wks mice the asymptomatic and pre-symptomatic stages respectively of NPC cerebellar disease.

To establish a correlation between asymptomatic, pre-symptomatic and symptomatic stages with PC loss in this mouse strain, PCs labeled with the fluorescent “nissl” staining NeuroTrace were quantified in the first 4 lobes of the anterior cerebellum, the cerebellar region that is affected the earliest in the NPC disease^31^, at 4, 8 and 12 wks (Fig. 1e). In *Npc1*^*nmf164*^ mice, we found significant loss of PCs at 8 (~50%) and 12 wks (~80%) when compared to WT mice, where PC density was not different between ages (Fig. 1e,f). More severe loss of PCs was found in male than in female mice (Supp. Fig. S2f). At 4 wks no changes in the density of PCs were found between the *Npc1*^*nmf164*^ and WT mice (Fig. 1f). Therefore, we designated 4, 8 and 12 wks as the early (asymptomatic), moderate (pre-symptomatic) and severe (symptomatic) stages of PCs degeneration respectively.

### Microglia activation occurs early in the cerebellar molecular layer of *Npc1*^*nmf164*^ mice

**Figure 2:**
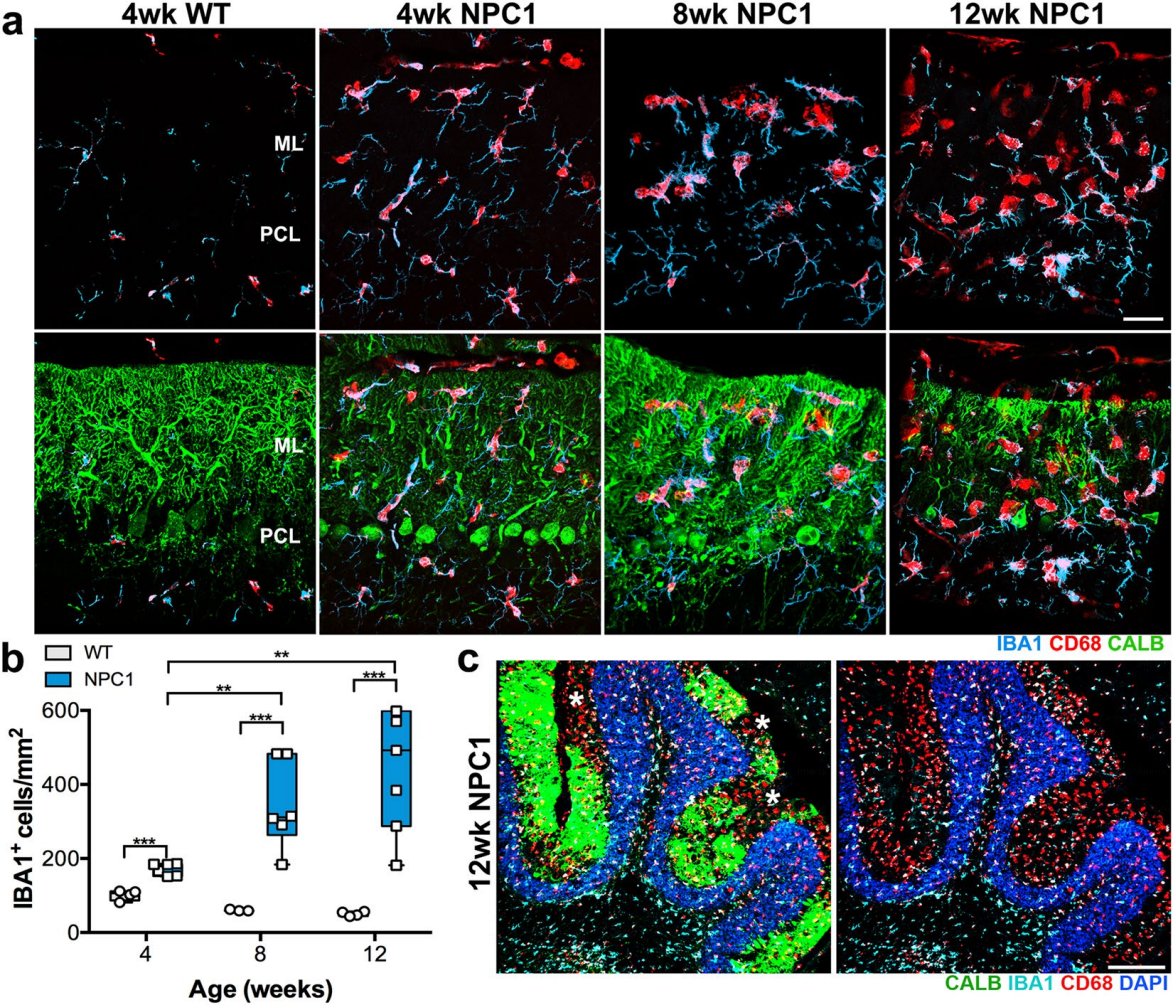
Pathological changes in microglia are found early and exacerbated with age in the *Npc1*^*nmf164*^ mouse. (**a**) Representative images from 4, 8, and 12 weeks old mice showing IBA1^+^ microglia cells (cyan) containing CD68^+^ phagosomes (red) at the ML where CALB^+^ dendrites from PC (green) reside. Changes in microglia morphology, and in the density and content of phagosomes, were already evident in 4 weeks old *Npc1*^*nmf164*^ mice and exacerbated at later stages when compared to WT mice. (**b**) The density of IBA1^+^ microglia were increased at 4, 8 and 12 weeks of age in *Npc1*^*nmf164*^ mice when compared to WT mice (n = 3–4 per age). (**c**) Representative images of anterior cerebellar lobules immunostained with CALB, IBA1 and CD68, showing regional loss of PCs, and massive invasion of microglia cells at the ML in a 12 weeks old *Npc1*^*nmf164*^ mouse. Data are presented as mean ± SEM n = 6–7. **P < 0.01, and ***P < 0.001. Scale bars: (**a**) 30 μm and (**c**) 200 μm.

Since microglial activation is a hallmark of NPC disease, we decided to study microglia in *Npc1*^*nmf164*^ mice at the different stages of the disease to establish the timing of microglial activation in correlation to PCs degeneration and behavioral deficits. Significant changes in microglia were evident in the *Npc1*^*nmf164*^ mouse cerebella especially at stages where significant loss of PCs was found. The density of IBA1^+^ cells at the ML was increased early and through the progression of PCs degeneration in *Npc1*^*nmf164*^ mice when compared to age-matched WT (Fig. 2a,b). In *Npc1*^*nmf164*^ mice, the density of microglia was significantly higher at 8 and 12 wks when compared to 4 wks mice (Fig. 2a,b). No significant differences in microglia density were found between WT mice (Fig. 2b). Accumulation of CD68^+^ phagosomes in microglia was also noticeable at all stages in *Npc1*^*nmf164*^ mice (Fig. 2a). We found it intriguing that even in the absence of PC loss, CD68 immunoreactivity in microglia at the ML (where PC dendrites reside) was so remarkable at 4 wks in *Npc1*^*nmf164*^ mice when compared to WT (Fig. 2a). In addition, at the severe stage of PCs degeneration, where the degeneration of PCs was evidently regional (CALB^+^-PCs), the activation of microglia in the ML was uniform through all the cerebellar anterior lobules (Fig. 2c), suggesting that microglial activation precedes the death of PCs.

**Figure 3:**
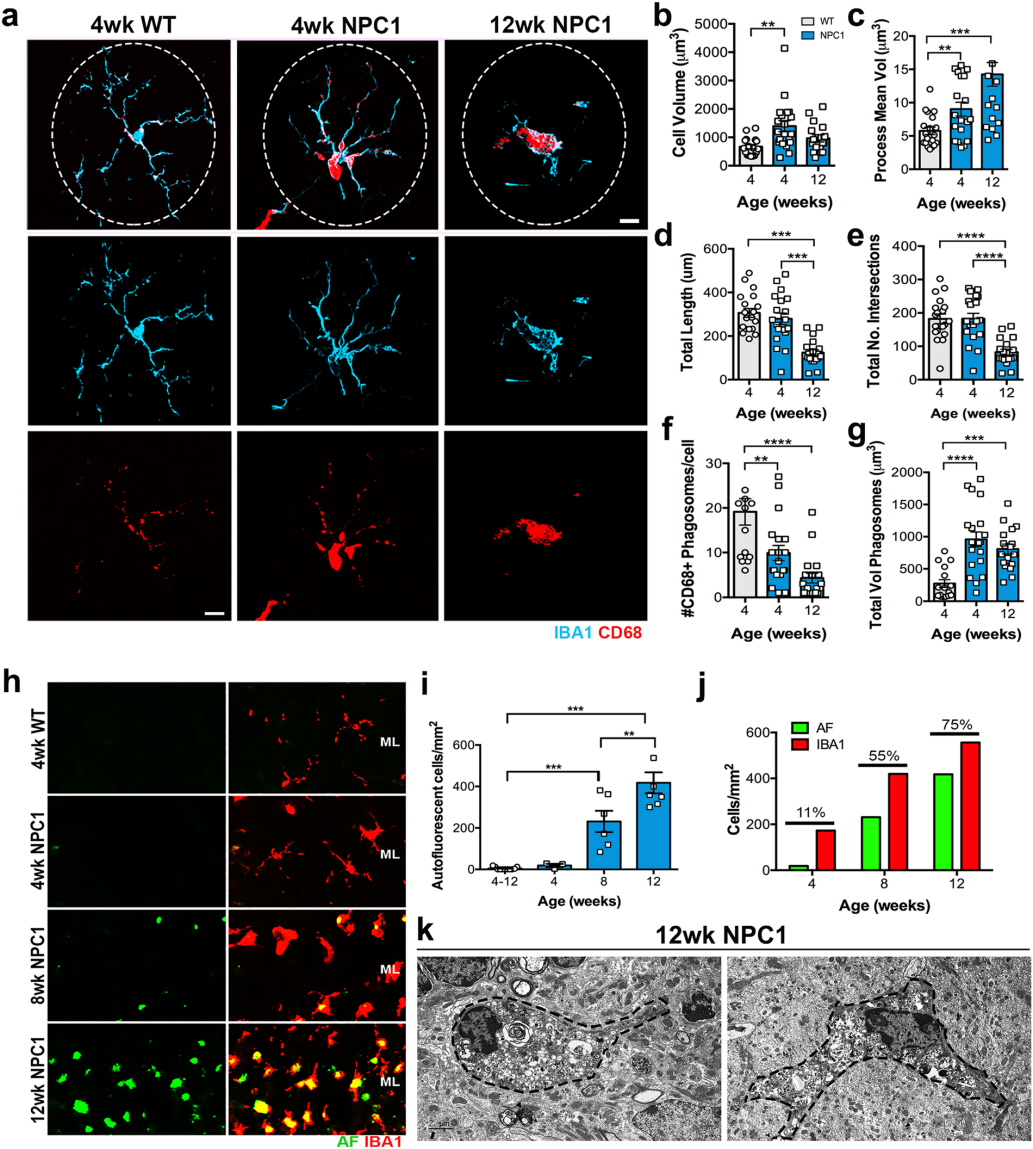
Morphological changes and accumulation of phagosomes in *Npc1*^*nmf164*^ IBA1^+^ microglia. (**a**) IBA1 (cyan) and CD68 (red) immunolabeled microglia from 4 and 12 weeks old *Npc1*^*nmf164*^ mice showed remarkable changes in morphology and phagosomes distribution in the cells when compared to WT microglia. Quantitative analysis of cell volume (**b**) showed an increased cell volume at 4 weeks of age in *Npc1*^*nmf164*^ mice, while the processes mean volume (**c**) was increased at 4 and 12 weeks of age in *Npc1*^*nmf164*^ mice when compared to WT mice. The total length (**d**) and number of intersections (**e**) of the microglia processes showed a significant decrease at 12 weeks of age in *Npc1*^*nmf164*^ mice, indicating the loss of cell ramifications. The number of CD68^+^ phagosomes (**f**) was decreased in *Npc1*^*nmf164*^ mice, but it is because of the accumulation of these phagosomes in the cell body, which create phagosomes with a significant larger average volume (**g**). (**h**) Autofluorescent cells (green) at the ML of *Npc1*^*nmf164*^ mice colocalized with IBA1 immunostaining (red) at 8 and 12 weeks of age. (**i**) The density of autofluorescent cells in the ML was significantly higher at 8 and 12 weeks of age. (**j**) The percentages of IBA1^+^ cells that were autofluorescent in *Npc1*^*nmf164*^ mice at 4, 8 and 12 weeks of age were 11%, 55% and 75% respectively. (**k**) Electron micrographs of phagocytic microglia in 12 wks old *Npc1*^*nmf164*^ ML. Data are presented as mean ± SEM, n = 4–7. *P < 0.05, **P < 0.01, ***P < 0.001. Scale bars: (**a**) 10 μm, (**h**) 20 μm, (**k**) 2 μm.

Morphological changes, such as increased size of the cell body, along with thicker and retracted processes, characterize microglia activation^32^. Since, no morphological changes were evident between WT microglia at the different time points analyzed in this study, quantitative analysis of morphological changes in *Npc1*^*nmf164*^ IBA1 and CD68 immunostained microglia at 4 and 12 wks were compared to 4 wks-WT microglia (Fig. 3a). The total cell volume of IBA1^+^ cells was significantly increased in *Npc1*^*nmf164*^ mice at only 4 wks when compared to WT mice (Fig. 3b). However, the mean volume of the microglial processes was significantly higher at both 4 and 12 wks in *Npc1*^*nmf164*^ mice when compared to WT (Fig. 3c). Total length of processes (Fig. 3d) and total number of intersections (Fig. 3e), showed a significant reduction in microglia ramification only at 12 wks in *Npc1*^*nmf164*^ mice, when ameboid shape cells were more evident (Fig. 3a). CD68 is a lysosomal protein that is commonly used as a marker for activated phagocytic myeloid cells^33,34^. Compared to WT, the number of CD68-phagosomes was significantly reduced at 4, and 12 wks in *Npc1*^*nmf164*^ mice (Fig. 3f). However, the average size of the CD68-phagosomes at both ages was significantly increased in *Npc1*^*nmf164*^ mice (Fig. 3g), suggesting that *Npc1*^*nmf164*^ microglia are actively phagocytic and unable to resolve their phagosomes.

The increased accumulation of CD68-phagosomes in the *Npc1*^*nmf164*^ microglia at the ML could be a reasonable consequence of unresolved lysosomes due to the deficiency of NPC1. However, it is interesting that these phagosome accumulations appear to be increased in the microglia of the ML, parallel to the degeneration of the PCs, suggesting that these cells could be actively phagocytosing the PC dendrites. Concomitant with this hypothesis, we found increased number of IBA1^+^ microglia with accumulation of autofluorescent material in their cell bodies at 8 and 12 wks in *Npc1*^*nmf164*^ mice (Fig. 3h,i). We found that 55% and 75% of IBA1^+^ cells at 8 and 12 wks respectively were accumulating autofluorescent material (Fig. 3j). The degree of PCs degeneration in the cerebellum at these stages corresponded well to the number of IBA1^+^ cells containing autofluorescent material in the ML of *Npc1*^*nmf164*^ mice (Fig. 3h–j). No autofluorescent cells were found in the WT mice and in 4 wks *Npc1*^*nmf164*^ mice. At the ultrastructural level, accumulation of unresolved phagosomes, multilamellar and multivesicular bodies, containing debris and lipids were also evident in 12 wks microglia in *Npc1*^*nmf164*^ mice (Fig. 3k), confirming the phagocytic activity of these cells at the ML.

### Cerebellar resident myeloid cells are increased in the ML of *Npc1*^*nmf164*^ mice

**Figure 4:**
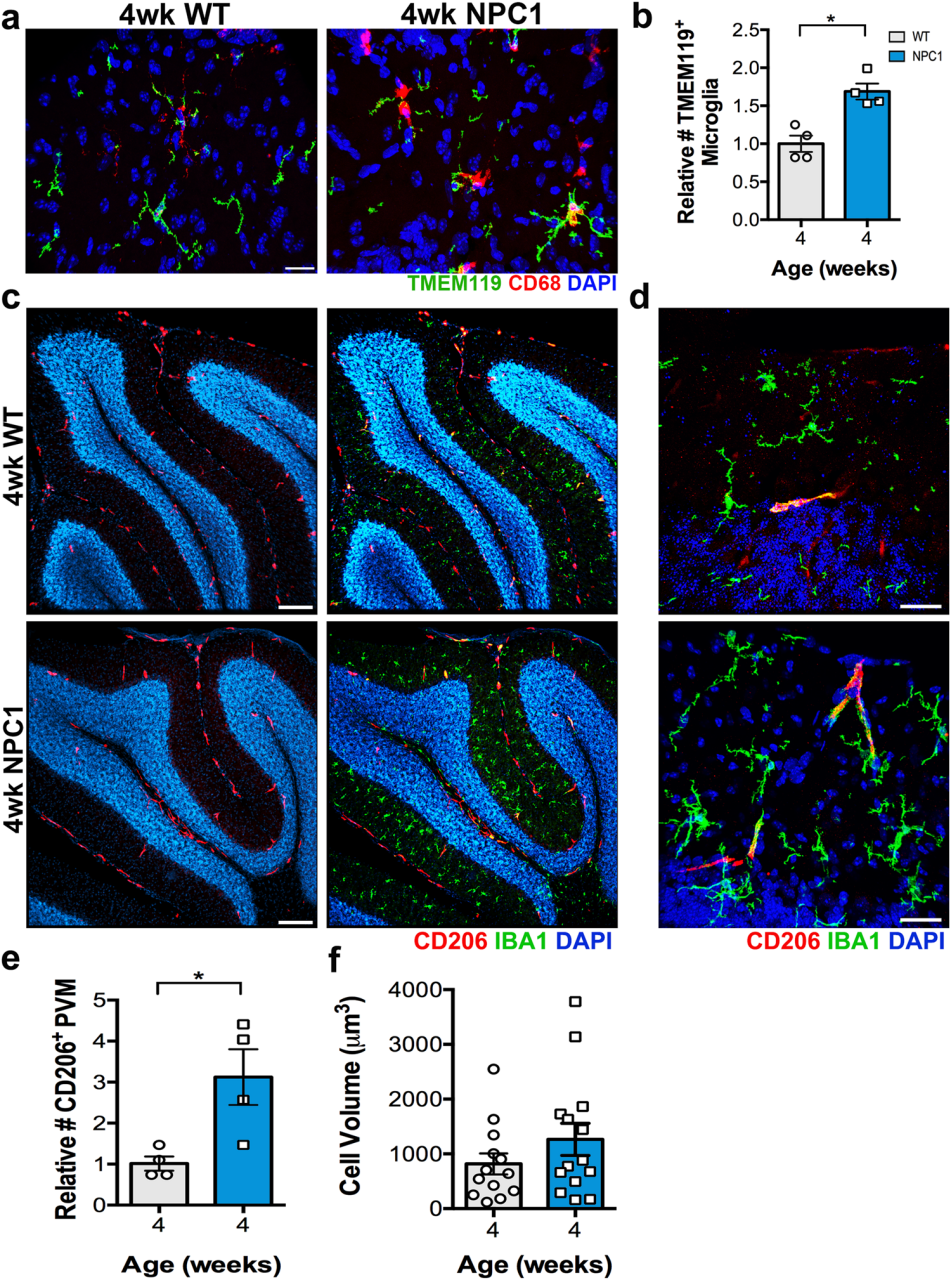
In *Npc1*^*nmf164*^ mice, the densities of resident microglia and resident perivascular macrophages are increased at early stage of NPC disease. (**a**) Resident microglia at 4 weeks of age were immunopositive for TMEM119 (green) and CD68 (red) at the ML in WT and *Npc1*^*nmf164*^ mice. (**b**) The number of TMEM119^+^ microglia was almost two times higher in *Npc1*^*nmf164*^ mice than in WT mice. (**c**) Low magnified images showing the scattered number and distribution of CD206^+^ resident perivascular macrophages in the cerebella of 4 weeks old WT and *Npc1*^*nmf164*^ mice. Not all the cells were IBA1^+^. The majority of CD206^+^ cells were observed at the meninges and at the white matter region of the cerebellum. (**d**) High magnified images of the ML showing perivascular macrophages that are positive for CD206 and IBA1 immunostaining at 4 weeks of age in WT and *Npc1*^*nmf164*^ mice. (**e**) Quantitative analysis of CD206/IBA1^+^ perivascular macrophages showed a significant increase in the ML of 4 weeks *Npc1*^*nmf164*^ mice. (**f**) No significant changes in the cell volume of CD206 cells between WT and *Npc1*^*nmf164*^ mice. Data are presented as mean ± SEM, n = 4 per group, *P < 0.05. Scale bars: (**a**) 20 μm, (**c**) 150 μm, (**d**) 20 μm.

Recently, a different mouse model of NPC (*Npc1*^*nih*^) was reported to have no evidence of infiltration of peripheral monocytes during the progression of NPC^20^. To determine if the increased population of IBA1^+^ cells in *Npc1*^*nmf164*^ mice were resident microglia, the TMEM119 antibody (a specific marker for this population of cells^35^) was used to quantify resident microglia in 4 and 8 wks WT and *Npc1*^*nmf164*^ mice. TMEM119 was present in microglia at 4 wks of age in WT and *Npc1*^*nmf164*^ mice, but absent in 8 wks *Npc1*^*nmf164*^ microglia, suggesting downregulation of this protein in *Npc1*^*nmf164*^ activated microglia with age. This finding agreed with recent reports that showed that microglia lineage markers are decreased in NPC1 deficient microglia^20^, supporting the possibility that a global decrease of microglia lineage genes is also occurring in *Npc1*^*nmf164*^ mice. At 4 wks, the number of TMEM119^+^ microglia were almost two times higher in *Npc1*^*nmf164*^ mice than in age-matched WT mice (Fig. 4a,b), indicating that the number of resident microglia is increased early in *Npc1*^*nmf164*^ mice. To identify the myeloid cells that were phagocytic but negative for TMEM119, cerebellar sections immunostained with TMEM119 were coimmunostained with CD68 (Fig. 4a). A small population of cells positive for CD68 but negative for TMEM119 were observed. Since it is known that a population of perivascular macrophages (PVM) are also permanent residents of the brain^36^, we decided to analyze if the number of these cells was also altered in *Npc1*^*nmf164*^ mice at 4 wks. To identify the PVMs in the cerebellum we used the CD206 antibody, which labels these specific population of macrophages and some endothelial cells^36^. CD206 was used in conjunction with the IBA1 antibody to discriminate macrophages from endothelial cells, only cells positive for both markers were quantified. CD206 immunostaining was found in few cells at the ML, and along the meninges of the cerebellum in both WT and *Npc1*^*nmf164*^ mice at 4 wks (Fig. 4c,d). The majority of these cells co-expressed the IBA1 protein and were identified as PVMs during our quantifications. We found that this population of CD206^+^/IBA1^+^ cells was also significantly increased in *Npc1*^*nmf164*^ mice (Fig. 4d,e). No significant changes in PVMs cell volume were found between WT and *Npc1*^*nmf164*^ mice (Fig. 4f). Overall, we found that both populations of resident myeloid cells in the ML, microglia and PVMs, were augmented in *Npc1*^*nmf164*^ mice.

### Microglia increase contacts with PC dendrites in *Npc1*^*nmf164*^ mice

**Figure 5:**
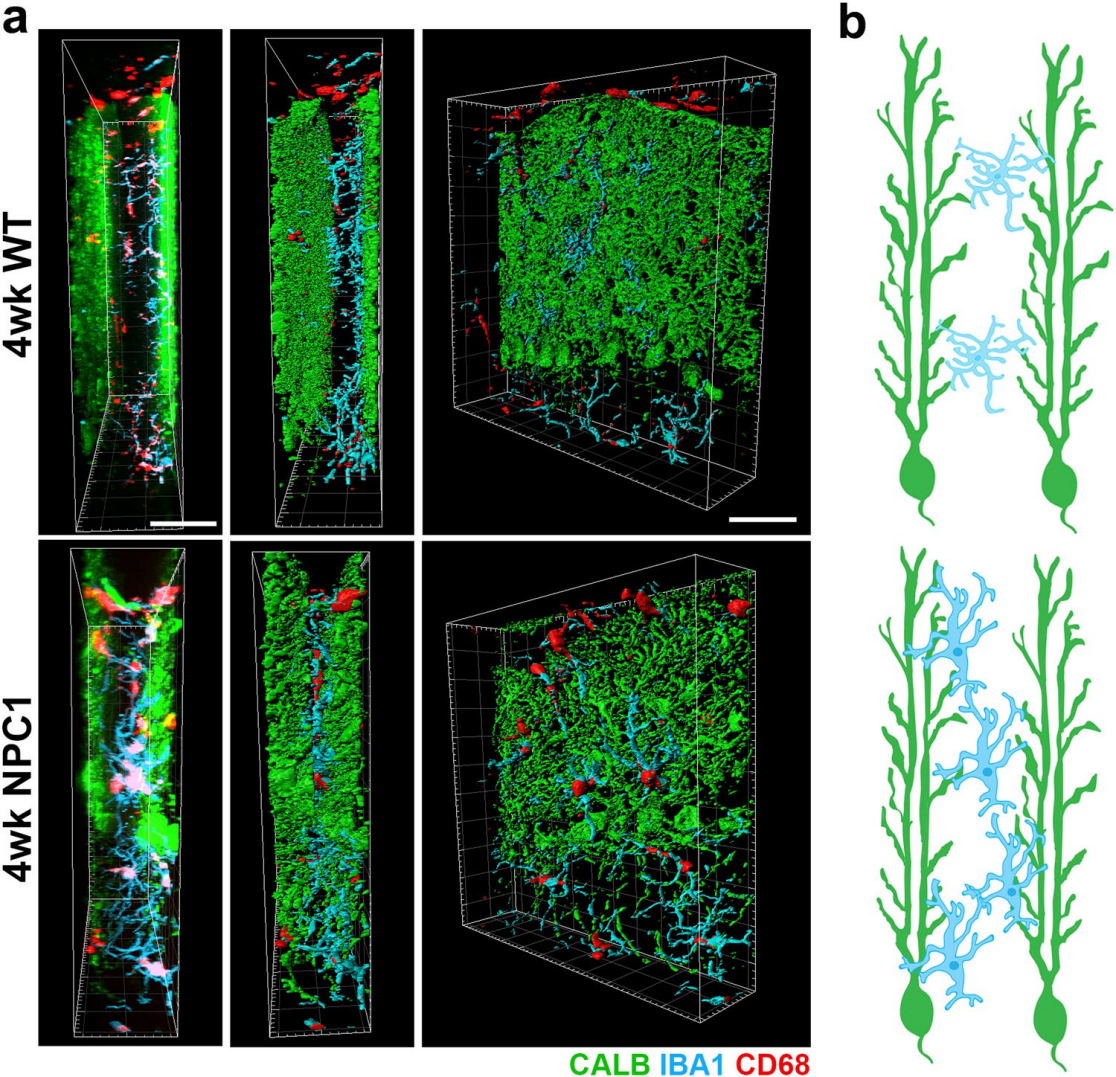
Microglia at the ML reside in the medio-lateral space between single monoplanar PC layers (PCL). (**a**) Medio-lateral views of 3D confocal and 3D surface rendering images of the ML from WT and *Npc1*^*nmf164*^ mice immunostained with IBA1(cyan), CD68 (red) and CALB (green). The PCL of the left was removed in the surface rendering Images of the third column to show the location of microglia (red and cyan) relative to the PCL at the right (green). (**b**) Representative diagram of the microglia location and distribution when observed from a medio-lateral view. At 4 weeks of age, more overlapping of activated microglia with the PCL was observed in *Npc1*^*nmf164*^ mice. Scale bars: 40 μm.

**Figure 6:**
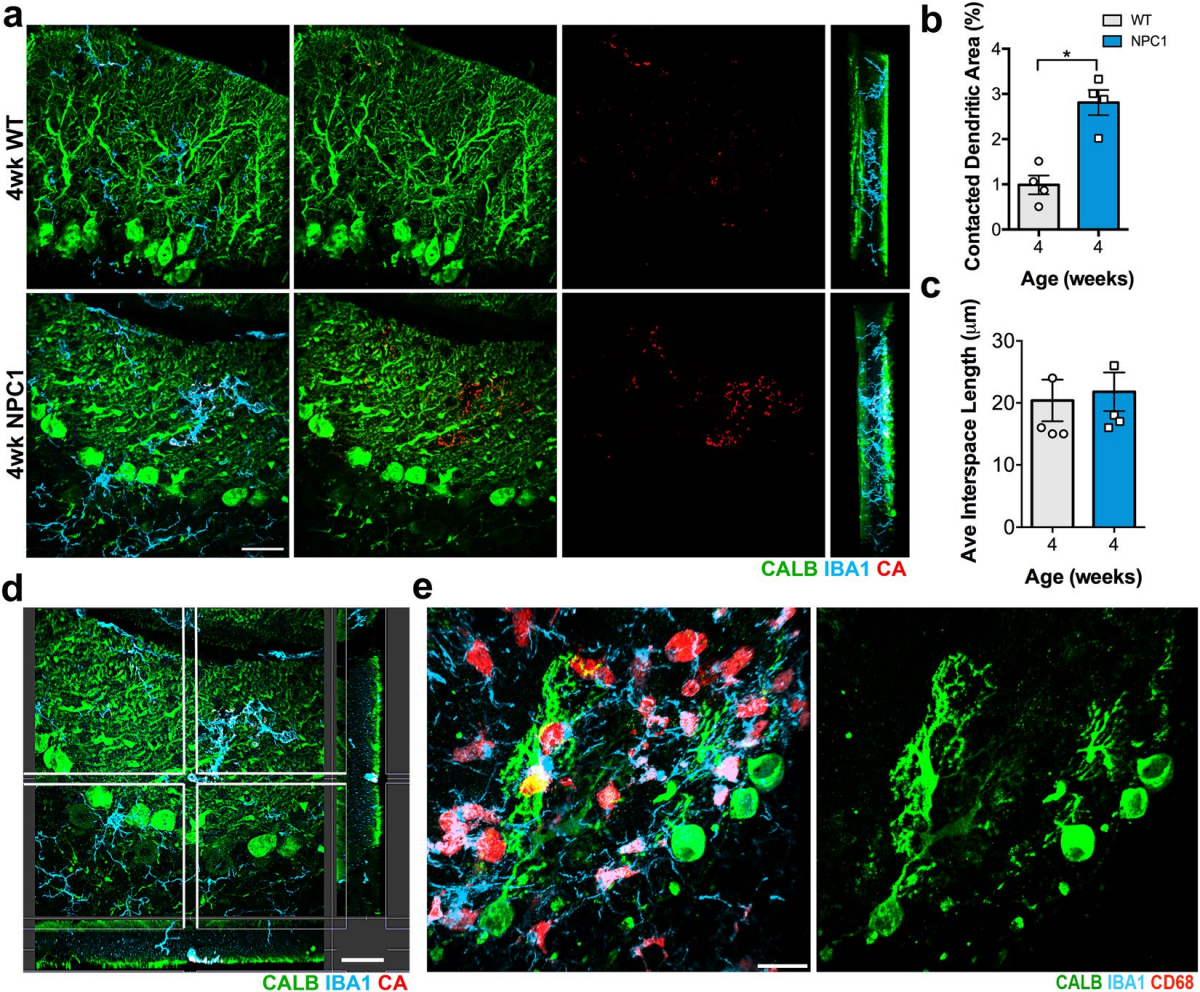
Microglial contacts and interactions with PC dendrites are increased at early stage of NPC disease in *Npc1*^*nmf164*^ mice. (**a**) Using the Imaris software, the regions of the CALB^+^ dendrites (green) contacted or wrapped by microglia (cyan) were segregated and pseudo-colored in red and named contacted area (CA). The CA was evidently increased in 4 weeks old *Npc1*^*nmf164*^ PC dendrites when compared to WT. (**b**) Quantitative analysis of the percentage of the PC dendritic area contacted by microglia at 4 weeks of age showed a significant increase in *Npc1*^*nmf164*^ mice when compared to WT mice. (**c**) No changes in average length of the space between single PC layers were found between WT and *Npc1*^*nmf164*^ mice. (**d**) Confocal 3D reconstruction of the 4 weeks old *Npc1*^*nmf164*^ sample presented in (**a**), showing the interaction of a microglia process with PC dendrites. Scale bar: 20 μm. (**e**) Images showing CALB^+^ PC with degenerating dendrites and activated microglia (IBA1^+^/CD68^+^) invading and engulfing them in a 12 weeks old *Npc1*^*nmf164*^ mouse. All data are presented as mean ± SEM, n = 4 per group, *P < 0.05. Scale bars: 20 μm.

A uniqueness of PCs is their elaborated and monoplanar dendrites, which are arranged as single layers spaced in between from each other, when observed from the mediolateral axis, to prevent intermingling of dendrites^37^. While assessing our immunostained cerebellar sections (with IBA1, CD68 and CALB), we noticed that microglial cell bodies were always detected scattered throughout the interspace between the single layers of PCs (Fig. 5a). 3D computing models from 3D images, confirmed that microglial cell bodies were mainly located at the interspace between the monoplanar PC layers in both 4 wks-WT and -*Npc1*^*nmf164*^ mice. In *Npc1*^*nmf164*^ mice, we noticed that, in addition to the increased density of microglia (Fig. 2), there was a noticeable closer proximity of microglia to PC dendritic layers when compared to the WT mice (Fig. 5a,b). To asses microglia interactions with PC dendrites, we used a 3D surface rendering tool (see methods) to quantify the percentage of CALB^+^-dendritic area in contact with or wrapped by, microglia processes in WT and *Npc1*^*nmf164*^ mice at 4 wks of age (Fig. 6a). While less than 1% of the measured CALB^+^-dendritic area was contacted by microglia in WT cerebella, almost 3% of the measured CALB^+^ dendritic area was contacted by microglia in *Npc1*^*nmf164*^ mice (Fig. 6b). Further, these microglia appeared activated as described before (Fig. 3). This interaction of microglia processes and bodies with PC dendrites was not the result of shrinkage of the interspace between PC single layers, as we found that the thickness of this interspace ranged between 15 to 35 μm in both WT and *Npc1*^*nmf164*^ mice (Fig. 6c). These findings, including the increased number CD68-phagosomes in microglia, suggest that the increased interaction of microglia processes with PC dendrites in the *Npc1*^*nmf164*^ mouse was due to the active phagocytosis of probable dysfunctional or degenerating dendrites by microglia (Fig. 6d). In support of this, CALB^+^-PC somata with few degenerated or no dendrites were often surrounded and contacted by a large number of activated microglia, a phenomenon that was more common in severe compared to earlier stages of the disease in *Npc1*^*nmf164*^ mice (Fig. 6e).

### Western diet exacerbated microglial activation and increased degeneration of PC dendrites in *Npc1*^*nmf164*^ mice

**Figure 7:**
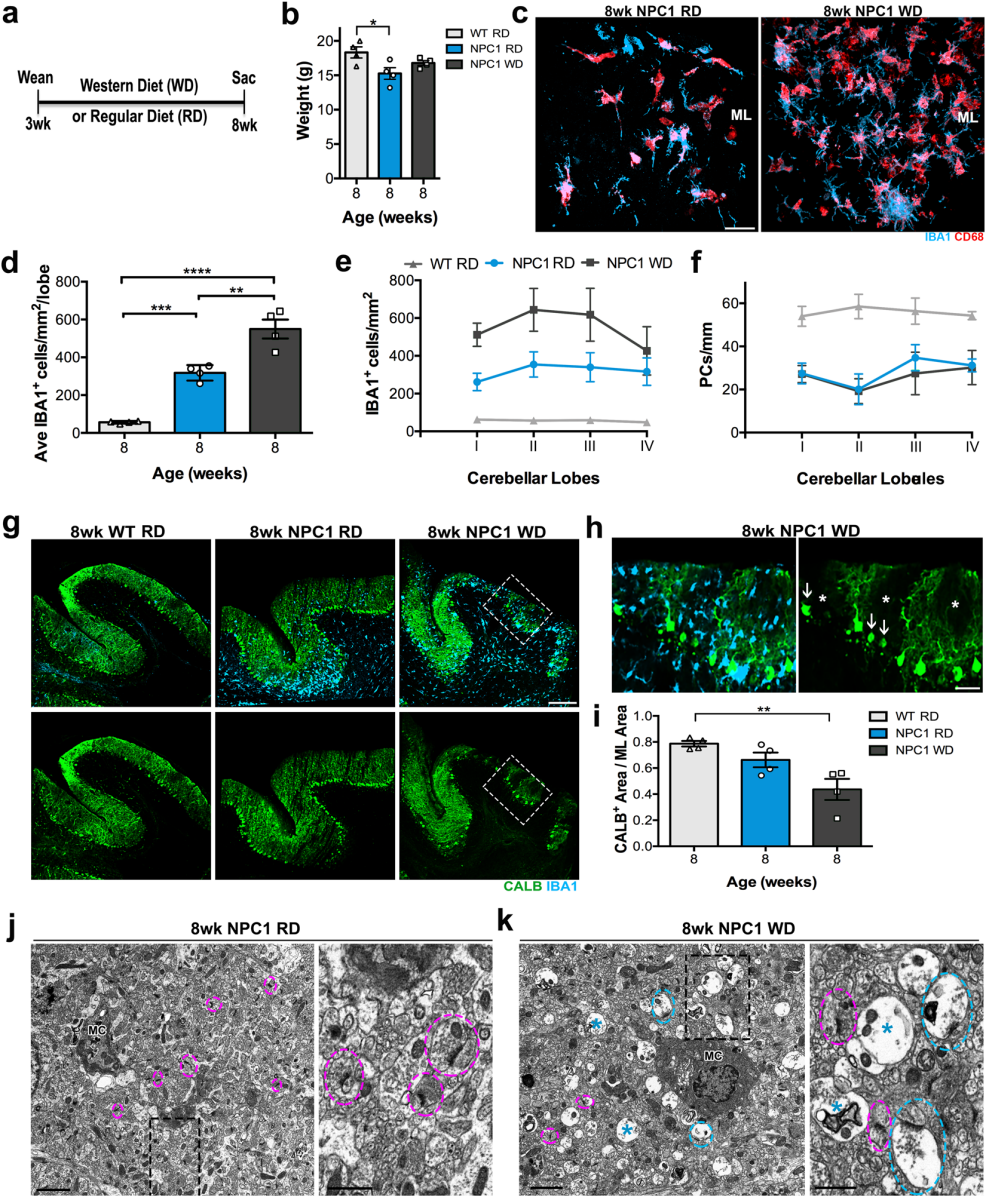
*Npc1*^*nmf164*^ mice fed a WD have increased density of microglia and a more severe loss of PC dendrites at 8 weeks of age. (**a**) WT and *Npc1*^*nmf164*^ mice were fed a WD or RD from wean (3 wks) for 5 weeks, and euthanized at 8 weeks of age. (**b**) The average weight of *Npc1*^*nmf164*^ RD mice, was slightly less (but significant) than WT RD mice at 8 weeks of age. (**c**) Microglia density at the ML was evidently increased in *Npc1*^*nmf164*^ WD mice when compared to *Npc1*^*nmf164*^ RD. (**d**) Quantitative analysis confirmed that the average of microglia density per lobule was significantly increased in *Npc1*^*nmf164*^ WD mice when compared to RD-fed mice. (**e**) Density of microglia per cerebellar lobule (I to IV) in *Npc1*^*nmf164*^ mice fed the RD or WD compared to WT RD mice. (**f**) Density of PC per cerebellar lobule (I to IV) in *Npc1*^*nmf164*^ mice fed the RD or WD compared to WT RD mice. (**g**) Low-magnified images showing cerebellar lobules I and II from WT RD, *Npc1*^*nmf164*^ RD and *Npc1*^*nmf164*^ WD cerebella immunostained with CALB (green) and IBA1 (cyan). (**h**) High magnified images from inserts in (**g**) showing the severity of CALB^+^ dendrites degeneration in *Npc1*^*nmf164*^ mice fed the WD. The (*) symbols show spaces where CALB^+^ dendrites have degenerated, arrows show PC somata without dendrites. (**i**) Quantitative analysis of the ratio between CALB^+^ area to the total area of the ML showed a significant decrease in the cerebellar lobules I and II from *Npc1*^*nmf164*^ WD mice when compared to WT RD mice. (**j**) Electron micrograph and magnified inset showing microglia process (MC) and normal synapses (pink circles) in *Npc1*^*nmf164*^ RD. (**k**) Transmission electron micrograph and magnified inset showing microglial cell (MC) and swollen presynaptic terminals forming synapses (blue circles) or not (blue *) in *Npc1*^*nmf164*^ WD. Data are presented as mean ± SEM, n = 4 per group, *P < 0.05, **P < 0.01, ***P < 0.001. Scale bars: (**d**) 30 μm, (**g**) 150 μm, (**h**) 40 μm, (**j**,**k**) 2 μm, insets 1 μm.

**Figure 8:**
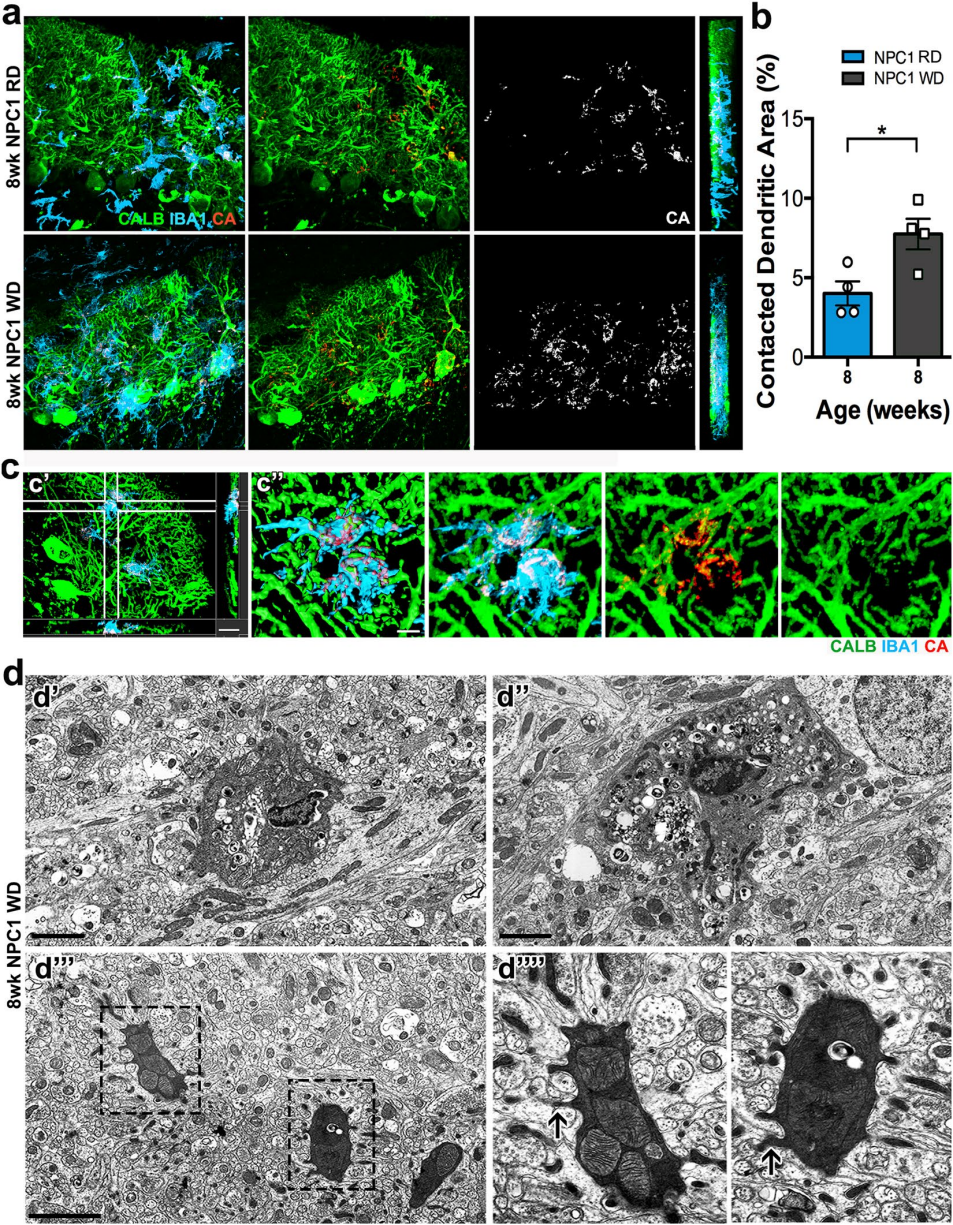
Microglial contacts and interactions with PC dendrites are increased in *Npc1*^*nmf164*^ WD mice. (**a**) Using the Imaris software, the regions of the CALB^+^ dendrites (green) contacted or wrapped by microglia (cyan) were segregated and pseudo-colored in red and named contacted area (CA). The CA was evidently increased in *Npc1*^*nmf164*^ WD dendrites when compared to *Npc1*^*nmf164*^ RD mice. (**b**) Quantitative analysis of the percentage of the PC dendritic area contacted or wrapped by microglia at 8 weeks of age showed a significant increase in the *Npc1*^*nmf164*^ WD mice when compared to *Npc1*^*nmf164*^ RD mice. (**c**) Confocal 3D reconstructions of the 8 wk *Npc1*^*nmf164*^ WD sample image in (a) with only four segregated microglia showing the overlapped or contacted area (red, CA) between IBA1^+^ microglia (cyan) and the CALB^+^ dendrites (green). (c’) Confocal 3D reconstruction showing how microglia processes and cell bodies between white lines wrap and interact with CALB^+^ dendrites in a *Npc1*^*nmf164*^ WD mouse. c”) High magnified 3D surface rendering and images of microglia showed in (c’). (**d**) Transmission electron micrographs of phagocytic activated microglial cells and processes of dark microglia (d”’ and d”” insets) in *Npc1*^*nmf164*^ WD mice. Arrows in (d””) show synaptic terminals engulfed by dark microglia processes. Data are presented as mean ± SEM, n = 4 per group, *P < 0.05. Scale bars: (**a**) 20 μm, (c’) 15 μm, (c”) 5 μm, (**d**) 2 μm.

Our data suggest a correlation between phagocytic activity of activated microglia in the ML and the degeneration of dendrites in *Npc1*^*nmf164*^ mice. Therefore, we hypothesized that increasing microglia activity would accelerate PCs degeneration further in *Npc1*^*nmf164*^ mice. It is known that a WD can induce microglial activation that coincides with neuronal damage in aging and AD mouse models^38^. Therefore, to increase microglia activity, WT and *Npc1*^*nmf164*^ mice were fed a WD^38^ from 3 to 8 wks of age (Fig. 7a). A control group of littermate mice were fed the regular diet (RD). Since we determined that PC loss in *Npc1*^*nmf164*^ mice occurred after 4 wks of age, we presumed that feeding 3 weeks old mice a WD for 5 weeks would be sufficient to affect microglia activity and the degeneration of PCs. Only a small decrease in weight was found in *Npc1*^*nmf164*^ mice fed a RD when compared to WT mice fed a RD or WD, no significant weight differences were observed between *Npc1*^*nmf164*^ and WT mice fed a RD or WD (Fig. 7b, Supp. Fig. S3a). Also, 5 wks of the WD did not alter the weight of WT mice when compared to RD-WT mice (Supp. Fig. S3a). Next, we determined the densities of microglia and PCs in WT and *Npc1*^*nmf164*^ mice fed either the RD or WD. We found no significant differences in the density of microglia or PCs between WT-RD and WT-WD mice (Supp. Fig. S3b,c). Since no differences were found between WT-RD and WT-WD, all the experimental results presented here were compared to the WT-RD mice. In contrast to WT-WD mice, 8 wks *Npc1*^*nmf164*^-WD mice had a significantly higher average of activated microglia density per lobe in the ML when compared to WT-RD and *Npc1*^*nmf164*^-RD mice (Fig. 7c,d). Our quantitative analyses also showed that the increased average density of microglia in *Npc1*^*nmf164*^-WD was more significant in the ML of the first two lobules (I and II) of the anterior cerebellum (Fig. 7e). In contrast, no differences were found in the average of the linear density of PC per lobe between *Npc1*^*nmf164*^ mice fed the RD or WD (Fig. 7f). It was noticeable that *Npc1*^*nmf164*^-WD mice, had more regions in the ML with a complete absence of CALB immunostained dendrites when compared to *Npc1*^*nmf164*^-RD mice (Fig. 7g). In fact, in the regions that showed complete absence of CALB immunostaining, it was often observed PC somata (with simplified or none dendritic tree) and abundant numbers of activated microglia (inset Fig. 7h, white arrows and stars). Quantitative analysis of the CALB^+^ dendritic area in the ML at the cerebellar lobules I and II, showed no significant differences between WT-RD and *Npc1*^*nmf164*^-RD mice (Fig. 7g–i). However, a significant reduction in CALB^+^-dendritic area was found in *Npc1*^*nmf164*^-WD mice when compared to WT-RD mice, suggesting that the WD accelerated the degeneration of PC dendrites in the ML of *Npc1*^*nmf164*^ mice. Ultrastructural evidence of the ML showed a more severe pathology in the cerebellum of *Npc1*^*nmf164*^-WD mice when compared to WT-RD mice (Fig. 7k). Microglia processes in the ML of *Npc1*^*nmf164*^-RD mice and the presence of normal synapses (pre- and post-synaptic terminals with postsynaptic density, pink circles) were observed in *Npc1*^*nmf164*^-RD mice (Fig. 7k). However, phagocytic microglia cell bodies and many swollen presynaptic terminals with (blue circles) and without (star) postsynaptic density, containing also intracellular debris were evident in the ML of *Npc1*^*nmf164*^-WD mice (Fig. 7k). Next, we measured the percentage of CALB^+^-dendritic area that was contacted or wrapped by IBA1^+^ microglia in *Npc1*^*nmf164*^ mice that were fed the RD or WD (Fig. 8a). Note that the images in Fig. 8a are examples of ML regions with similar number of PCs, but with increased dendritic loss in the WD fed mice. Our results showed that a significant higher percentage of CALB^+^-dendritic area is contacted or wrapped by microglia in the *Npc1*^*nmf164*^-WD mice (Fig. 8a,b). The higher density of microglia in *Npc1*^*nmf164*^-WD mice appear to interact more with the dendrites than in *Npc1*^*nmf164*^-RD mice (Fig. 8a,b). Fragments of PC dendrites wrapped by IBA1^+^ microglia were also observed in *Npc1*^*nmf164*^-WD mice (Fig. 8c’,c”). Evidence of phagocytic microglia in the degenerating regions of the ML of *Npc1*^*nmf164*^-WD mice was also observed by electron microscopy (Fig. 8d, Supp. Fig. S3d). The severe pathology observed at the ultrastructural level in the *Npc1*^*nmf164*^-WD mice was similar to 12 wks *Npc1*^*nmf164*^-RD mice (Supp. Fig. S3d). Furthermore, processes of dark activated microglia wrapping synaptic terminals (Fig. 8d”’,d””, arrows) were found particularly in *Npc1*^*nmf164*^-WD mice, confirming a more severe ML pathology along with an increased activation of microglial cells when compared to *Npc1*^*nmf164*^-RD mice. Collectively, our results suggest that in addition to the phagocytosis of detached dendritic fragments, microglia are also engulfing PC dendrites even before degenerated fragments are released from the damaged PC. Although we cannot discard the possibility that the WD accelerated the degeneration of dendrites in a neuronal autonomous way, our data suggest that higher density of activated microglia in the WD group leads to the increased engulfment of dendrites before the loss of PC somata (Fig. 8d).

## Discussion

In the healthy brain, microglia play important roles in neuronal and synaptic development, adult synaptic plasticity, and regulation of neurogenesis^39^. However, during pathological conditions, neuroinflammatory activation of microglia is a common and important hallmark of neurodegenerative diseases^40^. In fact, microglia that actively respond to neuronal tissue damage and display a specific molecular profile during CNS neurodegenerative conditions have been recently classified as disease associated microglia (DAM)^40,41^. Unknown signals from degenerating neurons promote DAMs transition from a homeostatic state to a more proliferative and phagocytic state. In NPC, early neuroinflammation and microglia activation are found in mouse models of the early-onset disease^20,23^, as well as the expression of DAM associated genes^20^. Since the *Npc1*^*nmf164*^ mouse exhibits a late-onset and milder disease progression than some other NPC mouse models, we first established three main stages of PCs degeneration, early, moderate, and severe. Remarkably, changes in the number and morphology of myeloid cells (resident microglia and PVMs) at the ML in *Npc1*^*nmf164*^ mice were already found at the early stage and became more pronounced as the degeneration of PCs progressed. In *Npc1*^*nmf164*^ mice, it was also evident that microglia in the ML had transitioned to a DAM state since they were accumulating phagosomes and autofluorescent material as the degeneration of PCs was progressing, suggesting active phagocytosis.

A novel finding in our study was the anatomical location of microglia in the ML. In the healthy cerebellum, microglia were scattered throughout the space in between the single monoplanar layers of PCs, with little or no interactions with the PC dendrites. This little interaction of microglia with PC dendrites may be the result of postsynaptic structures being tightly wrapped by Bergmann glia (BG)^42^. Intriguingly, in *Npc1*^*nmf164*^ mice, BG presents an abnormal morphological differentiation and a defective function during development, suggesting possible alterations in the ensheathment of postsynaptic structures in PC^43^. These defective functional interactions between BG and PC postsynaptic structures during development, contribute to deficits in synaptic connectivity and developmental acquisition of motor skills^43^. Our work has shown that before the loss of PCs and right after postnatal development (4wks), microglia were closer to the PC monoplanar layers contacting/wrapping dendritic structures of these neurons, suggesting that these microglia were sensing changes in the dendrites that were attracting them. It is possible that the poor interaction between BG and PC dendrites caused the increased interaction of microglia with the postsynaptic dendrites, making them more susceptible to be phagocytosed by these cells. It is thought that “neurodegeneration-associated molecular patterns” (NAMPs), such as ATP gradients, released from injured or stressed neurons can induce the migration of microglia to these injured sites^40,44,45^. The interferon regulatory factor-8 gene (*Irf8*) is a transcription factor that has been identified as a regulator of microglial reactivity, motility, and chemotaxis^46,47^. In fact, genetic deletion of this gene affects the expression of chemotaxis associated genes and impairs nucleotide-induced chemotactic activities in microglia^46^. Interestingly, genetic deletion of *Irf8* in *Npc1*^*nih*^ mice, inhibits the activation of microglia, delays PCs degeneration and extends mice survival 15%^20^, suggesting that blocking activation and chemotactic activities in microglia prolongs PC survival in NPC. The early shift of microglia toward PC dendrites may indicate that the degeneration of dendrites is an early pathological event that promotes the proliferation, activation, and migration of microglia in the ML of *Npc1*^*nmf164*^ mice. Further research is needed to identify the pathological changes in dendrites that precede and contribute to PCs degeneration and the progression of NPC.

The involvement of activated microglia in the progression of different neurodegenerative diseases has been well documented^40^. In fact, microglia can also induce neuronal degeneration and death by engulfing and phagocytosing synapses, axons, myelin or/and dendrites from living neurons^18^. In this study, we found it very appealing to see PC somata with small or none dendritic trees at the severe stage of the NPC neurodegeneration. In addition, those particular spaces in the ML lacking PC dendrites were always crowded with activated microglia that were evidently still interacting and engulfing “dendritic debris”. Increasing evidence has demonstrated that microglia and monocytes deficient in the NPC1 protein have increased phagocytic activity^20,48^, supporting our observations of active wrapping or engulfment of PC dendrites by phagocytic microglia in the *Npc1*^*nmf164*^ mouse. So, our next question was whether or not increased activation of microglia by an additional neuroinflammatory stimulus, would increase the severity of PCs degeneration in the *Npc1*^*nmf164*^ mouse. To answer this question, WT and mutant mice were provided a WD that was developed by incorporating the majority of the dietary components found in an average diet of westernized countries, including high fat from animal products, high fructose corn syrup, and sucrose among others^38^. Previous studies have demonstrated that chronic consumption of this particular diet increases microglial activation, proliferation and phagocytic activity in aging and in an APP/PS1 mouse model^38,49^, by damaging the cerebrovascular unit, and increasing monocytes infiltration^50^. In the current study, 5 wks consumption of this WD did not alter microglia number nor the number of PCs in WT mice. However, it was evident that the WD amplified the extent of microglia activation in the ML of *Npc1*^*nmf164*^ mice. Although, the loss of PCs was similar between the *Npc1*^*nmf164*^ mice fed either the RD or WD, the extent and severity of dendritic degeneration were significantly exacerbated by the WD and it occurred along with excessive activation of microglia. In addition, we also observed PC somata lacking dendrites in *Npc1*^*nmf164*^ mice fed the WD, which could also contribute to the similar density of PC between the diet groups. However, the severity of the PC dendritic loss in *Npc1*^*nmf164*^-WD mice corresponded well to the increased interactions and engulfment of PC dendrites by activated microglia. In fact, the areas in the PC dendritic region contacted or wrapped by microglia were two times higher in the *Npc1*^*nmf164*^-WD group than in the mutant-RD group, and pathological changes in the ML were more severe in the WD-fed mice.

The mechanism by which certain diets induce neuroinflammation in the brain has been extensively studied^51^. Recent evidence is suggesting that obesity-induced neuroinflammation in diet-induced obesity models is actually caused by the diet itself^51^. Diets that are high in animal fat, sugars or both can cause activation of the peripheral innate immune system, the release of pro-inflammatory mediators into the circulation, alterations in the gut microbiome and neuroinflammation in the brain^51^. For instance, WD consumption has been linked to alterations in the microbiome that lead to cognitive impairment^52^. Furthermore, systemic inflammation and alterations of the gut microbiome can alter the microglia phenotype and the progression of CNS neurodegenerative conditions^32^. In our study, 5 wks of WD consumption were enough to increase microglia associated pathology in the absence of obesity in the *Npc1*^*nmf164*^ mouse. However, the deficiency of *Npc1* not only affects the brain but also induces additional systemic pathologies that include liver disease, splenomegaly and gastrointestinal inflammation^2,48,53^. These systemic pathologies in combination with the already activated state of microglia in the cerebellum during NPC may intensify the impact of WD in microglia activation in the *Npc1*^*nmf164*^ mouse. The fact that consumption of a WD significantly exacerbated the cerebellar pathology in the *Npc1*^*nmf164*^ mouse emphasizes the role of environmental factors in the progression of neurodegenerative diseases as demonstrated in other disease and aging models^54–56^. Collectively our data show that PC loss in the *Npc1*^*nmf164*^ mouse model is preceded by microglia activation and the engulfment of dendrites by these phagocytic cells, further studies are necessary to determine the functional consequences of these pathological interactions.

## Materials and Methods

### Animals

All experiments involving mice were conducted in accordance to policies and procedures described in the Guide for the Care and Use of Laboratory Animals of the National Institutes of Health and were approved by the Animal Care and Use Committees at the Rowan University School of Osteopathic Medicine and at The Jackson Laboratory. The C57BL/6J-*Npc1*^*nmf164*^/J mouse strain (Jax stock number 004817) was provided by Dr. Robert Burgess at The Jackson Laboratory. *Npc1*^*nmf164*^ heterozygous mice were bred and housed in a 12/12-hour light/dark cycle to generate both WT and *Npc1*^*nmf164*^ homozygous mutant mice. Both males and females were used in this study and the distribution of these mice are shown in Supplementary Figures. For the WD experiments, a cohort of WT and *Npc1*^*nmf164*^ mice were randomly assigned to the two different diets (3 females + 1 male per group) and were maintained from wean for 5 weeks on standard LabDiet^®^ 5K54 (referred as Regular Diet, RD) or TestDiet® 5W80 (WD), which was adapted from the TestDiet® 5TLN by adding high fructose corn syrup, lowering fiber, and increasing milk protein and animal fat^38^.

### Behavioral tests

#### Ladder rung walking task

The apparatus consisted of 20 metal rungs forming a ladder floor that is raised above the surface. Each mouse was placed at one end of the apparatus and videotaped from the side as the mouse walks-transversely across the ladder from the beginning to the end, investigators recording the video were blinded to the genotype of the mice. Two investigators blinded to the genotype of the mice, quantified misses and slips of the forelimbs or hindlimbs for each mouse. The test was repeated three times and results were summed and divided by 60 (3 X walk through the 20 metal rungs) and averaged between investigators.

#### Marble burying test

The burying of marbles in the cage by laboratory mice is considered a normal spontaneous behavior^30^. However, the lack of the behavior or the excess of it, can be indicative of changes in brain synaptic function. It has been shown that genetic manipulations, neurological diseases or head injury alter the marble burying behavior in mice when compared to control mice. Also, mice presenting a repetitive behavior will bury the highest percentage of marbles in a shorter time^29^. The marble burying test consisted in adding a mouse to a cage where 12 marbles were equidistant in a 3 × 4 arrangement over the surface of clean bedding. After 30 minutes, the mouse was removed from the cage and a picture of the cage with the marbles was taken. Investigators performing the test were blinded to the genotype of the tested mice. The number and percentage of marbles that were buried (to 2/3 their depth) after 30 minutes were calculated by an investigator that was blinded to the genotype.

#### Digging and wall rearing test

Digging behavior is a species-typical behavior observed in rodents^30^. Laboratory mice dig vigorously in deep bedding such as wood chips. The test consisted of placing the mouse in a cage filled ~5 cm deep with bedding. The mouse behavior was video-recorded in the cage for 3 minutes. After the 3 minutes the mouse was removed and placed back on its original cage. Investigators recording the videos were blinded to the genotype of the mice. The analysis of the mouse behavior during those 3 minutes included the number of digging actions, the total duration of digging^30^ and the total duration of wall rearing. Investigators that analyzed the video-recordings were blinded to the genotype of the mice.

### Mouse perfusion and tissue preparation

Mice were euthanized with CO_2_ and transcardially perfused with 1X PBS followed by 4% paraformaldehyde. After perfusion, mice were decapitated and their brains were carefully dissected and fixed by immersion in 4% paraformaldehyde overnight. After fixation, brains were rinsed in 1X PBS, immersed in 30% sucrose/PBS solution overnight at 4 °C, frozen in OCT, and cryosectioned at 30 μm or 50 μm (floating sections).

### Immunohistochemistry

For immunostaining, brain sections in slides (30 μm) or as floating sections (50 μm) were rinsed once in 1X PBT (PBS + 1% Triton 100X) and incubated in primary antibodies diluted with 1X PBT + 20% normal donkey serum for two nights at 4 °C. After incubation with primary antibodies, sections were rinsed three times with 1X PBT for 10 min and incubated for two hours in the corresponding secondary antibodies (1:800, Jackson-ImmunoResearch). Tissue was then washed three times with 1X PBT for 10–15 min, incubated with DAPI and mounted in Poly aquamount (Polysciences). The following primary antibodies were used: rabbit anti-IBA1 (1:200, Wako), mouse anti-CALB (calbindin,1:200, Sigma-Aldrich), rat anti-CD68 (1:200, Biorad), and rat anti-CD206 (1:200, Bio-Rad). To stain PC somata, the NeuroTrace™ 530/615 Red Fluorescent Nissl Stain was used (NT, 1:80, ThermoFisher Scientific).

### Microscopy image analysis

All the imaging and quantitative analyses were performed in the first four anterior cerebellar lobules (I-IV). For quantification of NT^+^ PC, IBA1^+^ cells, autofluorescent cells, TMEM119^+^ microglia, and CD206^+^ PVM at the ML of the cerebellum, four images (1 per lobule) were taken from two cerebellar cryosections for each mouse (8 images per mouse) with an inverted Leica DMi8 fluorescent microscope. The imaged regions were randomly selected and investigators were blinded to the genotype. Once the images were taken, a box of 160 μm × 205 μm was used to crop the images (2 boxes per image), so that the area used for the cell counting was consistent between images/animals, and included only the Purkinje cell layer (PCL) and the ML. The cropped images were manually counted using the cell counter plugin from the ImageJ (1.47 d) software. Investigators were blind to the genotype of the tissue while counting the cells.

For 3D image reconstructions and analyses, three sagittal 50 μm cerebellar sections were immunostained by free floating immunohistochemistry. All the images analyzed by the Bitplane Imaris software were acquired using a Nikon A1R Confocal System equipped with Live Cell 6 Laser Line and Resonant Dual Scanner. Confocal image stacks were acquired using a 63X objective lens with a 1 μm interval through a 50 μm z-depth of the tissue. Three confocal images per mouse were taken from the first three lobes (1 per lobe). Quantitative analysis of 3D microglia morphology was performed using the Surface rendering tool for cell volume and the Filament Tracer for processes volume and ramification, both tools are part of the Bitplane Imaris software. Confocal z-stack images of ~40 μm were taken and twenty IBA1^+^ or CD206^+^ cells (5 per mouse, n = 4 mice) were segregated using 3D surface rendering to be used for the Filament Tracer tool that determines processes length, volume and ramification. For 3D/4D visualization of high magnified confocal images from the cerebellar ML, the 3D surface rendering was also used to segregate IBA1^+^ microglia, CALB^+^ PC dendrites, and CD68^+^ phagosomes inside microglia. Images of the surface renderings at different angles (sagittal and coronal) to show microglia location in the ML were taken as snapshots using Imaris, and the distance between the single PC layers were also calculated from these surface renderings. To determine the percentage of the CALB^+^ dendritic area contacted by IBA1^+^ microglia, a surface of CALB^+^ dendrites in 10X magnified images was created in each sample analyzed, then the “Mask all” tool was used to select the areas that were overlapping between the CALB and IBA1 channels by clearing all the IBA1 fluorescence that was not found overlapping/contacting the CALB^+^ dendrites rendering surface. The IBA1 area calculated and provided by the software was divided by the CALB^+^ dendritic area and multiplied by 100 to determine the percentage. Three to four images per mouse (n = 4) were used for these quantifications. To quantify the area occupied by CALB^+^ dendrites in the ML of the two first cerebellar lobules (I and II), the ML was manually traced using a draw tool from Imaris and the total area was calculated and provided by the software. Then, the CALB^+^ PC and dendrites were selected by using surface rendering, and the CALB^+^ area was calculated and provided by the software. The ratio between CALB^+^ area and total ML area was determined from three images per mouse (n = 4 mice).

### Electron microscopy

After perfusion with 4% PFA and dissection, brains from RD and WD fed mice (8 wks) were hemisected in the midsagittal plane, one of the hemisections was fixed overnight (2% paraformaldehyde + 2% glutaraldehyde in diluted in 0.1 M cacodylate buffer with 0.05% CaCl2) for electron microscopy. Resin embedding of the tissue was performed as previously described^57^. Small pieces of the processed cerebella were infiltrated with 50/50 Epon-Araldite resin and propylene oxide for 1 h, then in 100% Epon-Araldite and left in the desiccator overnight. The next day the cerebellum samples were placed in cubic molds and embedded in 100% resin. The resin block was trimmed and, using an ultramicrotome (Sorvall MT-2), longitudinal sections were cut; semi-thin sections (1 μm thick) for light microscopy, and ultrathin (90 nm) for electron microscopy. For light microscopy, semi-thin sections were stained using methylene blue-azure II and basic fuschin. Thin sections were examined with a JEOL JEM-1011 electron microscope equipped with a Gatan digital camera (Model-832) to describe the ultrastructural features of the cerebellar ML.

### Statistical analysis

Data were analyzed using GraphPad Prism software. Significance was calculated using unpaired t tests for comparisons between two groups and one-way multifactorial analysis variance (ANOVA) followed by Tukey posthoc tests for multiple comparisons. p-values are provided as stated by GraphPad Prism software and significance was determined with p-values less than 0.05.

## Supplementary information

Supplementary Material
