# Supplementary material for: Increased interactions and engulfment of dendrites by microglia precede Purkinje cell degeneration in a mouse model of Niemann Pick Type-C
Source: Sci Rep. 2019 Oct 11;9:14722. doi: 10.1038/s41598-019-51246-1 (PMC6788982; doi:10.1038/s41598-019-51246-1)
Supplement: Supplementary file 1 — Supplementary Material [file 41598_2019_51246_MOESM1_ESM.pdf]

Supplementary Information

Increased interactions and engulfment of dendrites by microglia precede Purkinje cell degeneration in a mouse model of Niemann Pick Type-C

Larisa Kavetsky[1], Kayla K. Green[1], Bridget R. Boyle[1], Fawad A. K. Yousufzai[1], Zachary M. Padron[1], Sierra E. Melli[1], Victoria L. Kuhnel[1], Harriet M. Jackson[2], Rosa E. Blanco[3], Gareth R. Howell[2] and Ileana Soto[1*]

Supplementary Figures:

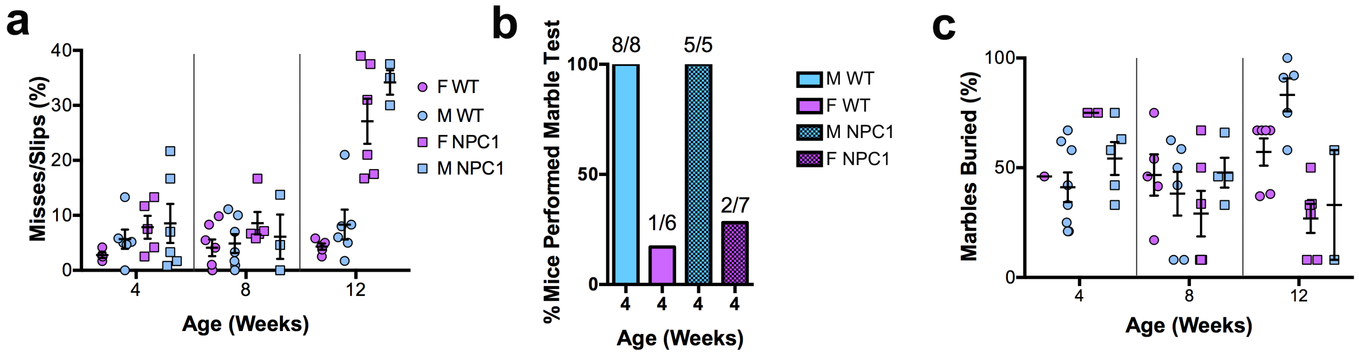

Fig.S1. a) Female and male mice distribution in the quantitative analysis of the ladder rung walking task. b) Percentage of male and female mice that performed the marble burying test at 4wks of age. c) Female and male mice distribution in the quantitative analysis of the marble burying test.

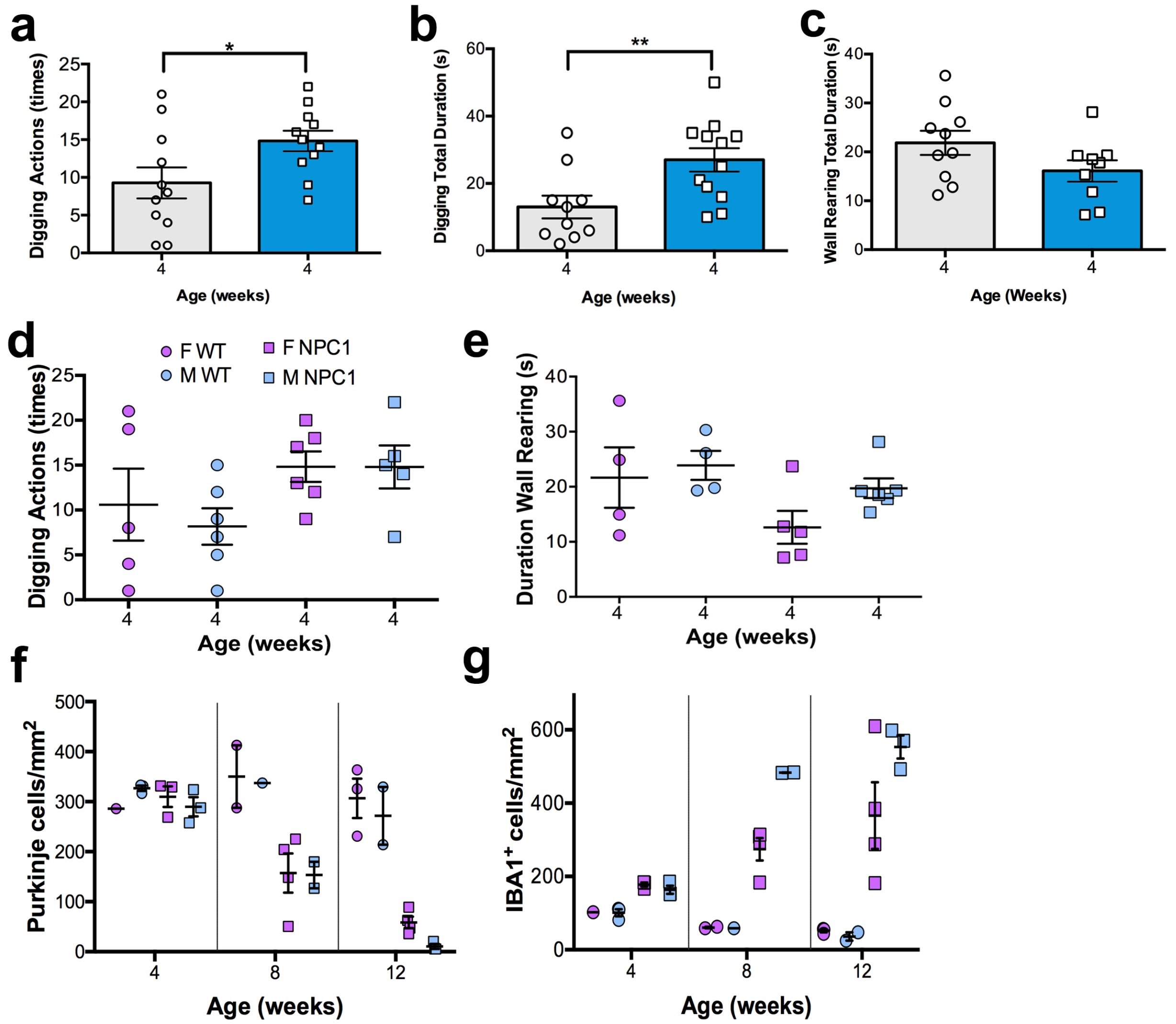

Fig. S2. a) Significant differences were found between WT and *Npc1^{nmf164}* mice in the number of burying actions at 4wks of age. b) The digging total duration was significantly higher in 4 weeks old *Npc1^{nmf164}* mice when compared to WT mice (n=10-12). c) No differences were found in wall rearing activity by 4wks WT and *Npc1^{nmf164}* mice. d-e) No differences were found between female and male in digging and wall rearing activity. f-g) Female and male mice distribution in quantifications of PCs and IBA1$^+$ cells between groups. *P < 0.05; ***P < 0.001.

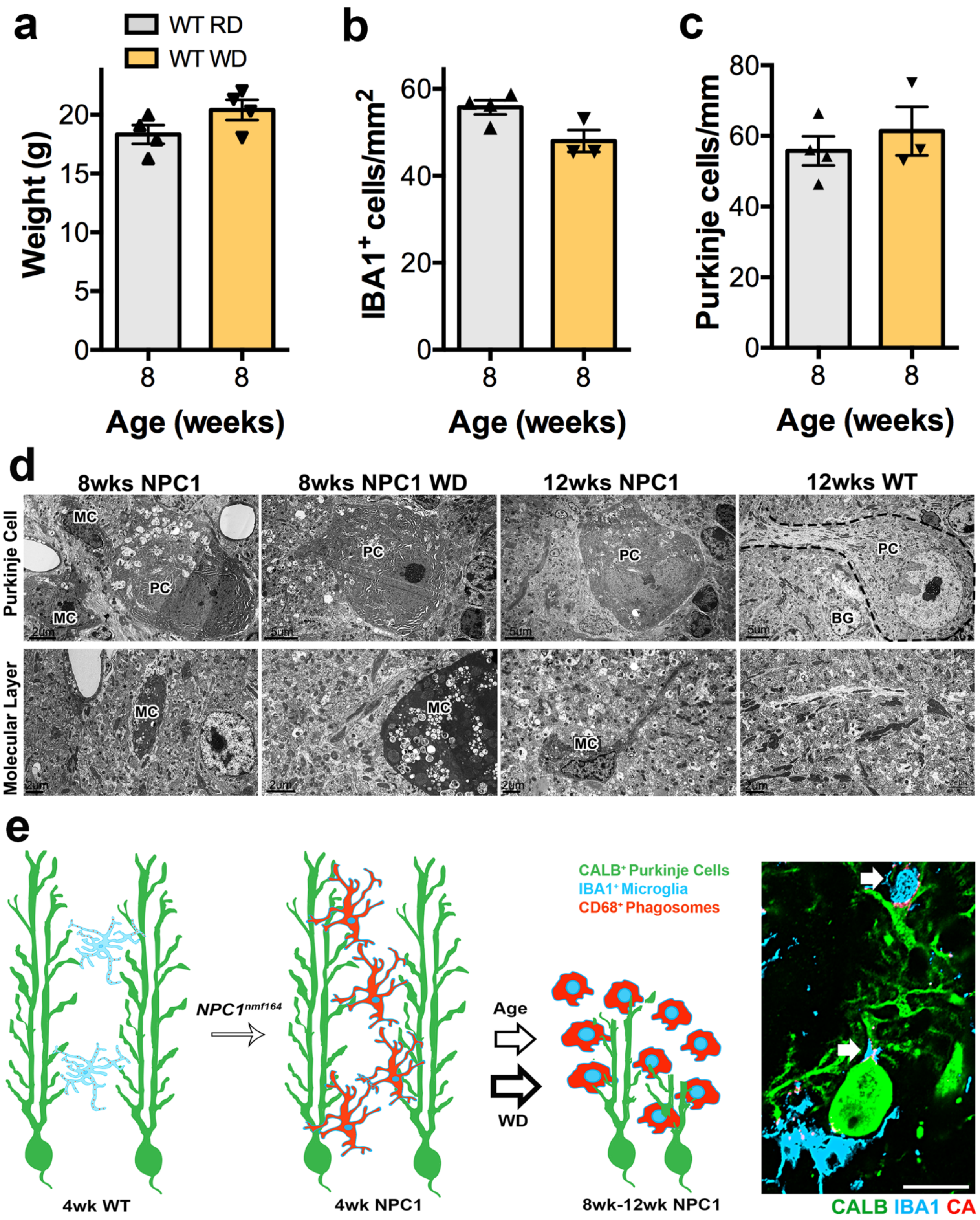
a
WT RD
WT WD
Weight (g)
Age (weeks)
b
IBA1+ cells/mm2
Age (weeks)
c
Purkinje cells/mm
Age (weeks)
d
8wks NPC1
8wks NPC1 WD
12wks NPC1
12wks WT
Purkinje Cell
Molecular Layer
MC
PC
BG
e
NPC1nmf164
Age
WD
CALB+ Purkinje Cells
IBA1+ Microglia
CD68+ Phagosomes
4wk WT
4wk NPC1
8wk-12wk NPC1
CALB IBA1 CA

Fig. S3. a) No differences in body weight were found between WT RD and WT WD mice. b) No differences in the density of microglia in the ML were found between WT RD and WT WD mice. c) No differences in the density of PCs were found between WT RD and WT WD mice. Data are presented as mean ± SEM, n=4 per group. d) Electron micrographs showing PCs and the ML from 8wks RD and WD-fed *Npc1*^{nmf164} mice, and 12wks RD *Npc1*^{nmf164} and WT mice. e) Schematic illustration of how the *Npc1*^{nmf164} mutation causes the early enlargement and displacement of microglia toward the PC dendrites concomitant with the accumulation of phagosomes (red). As NPC disease progresses the density of microglia and accumulation of phagosomes continue increasing along with the degeneration of PC dendrites. Consumption of a WD exacerbates the activation of microglia and the dendritic pathology in *Npc1*^{nmf164} mice.
